# Feasibility analysis of negative pressure wound therapy for odontogenic ascending necrotizing fasciitis: a single-center single-arm retrospective case series

**DOI:** 10.1186/s12903-026-09499-6

**Published:** 2026-08-10

**Authors:** Shu-Jun Chen, Ning Ji, Yu-Xuan Chen, Jian-Rui Xiao, Xiao-Zong Wei, Yan-Kun Liu, Lin-Wei Zhao

**Affiliations:** 1 Department of Stomatology, The 82nd Group Army Hospital of PLA, No. 991 Baihuadong Road, Baoding, Hebei Province 071000 P.R. China; 2 Department of Quality Control, The 82nd Group Army Hospital of PLA, Baoding, Hebei Province 071000 P.R. China

**Keywords:** Ascending necrotizing fasciitis, Necrotizing soft tissue infection, Odontogenic infection, Negative pressure wound therapy, Clinical feasibility

## Abstract

**Background:**

Odontogenic ascending necrotizing fasciitis (ANF) is an extremely rare, rapidly progressive, and destructive craniomaxillofacial necrotizing soft tissue infection. Advanced surgical drainage is essential to improve cure rates, shorten the disease course, and alleviate patient suffering.

**Methods:**

This study was designed as a single-center, single-arm, retrospective feasibility case series. A total of 15 consecutive patients diagnosed with odontogenic ANF and treated with standardized NPWT from March 2016 to March 2024 were enrolled. Uniform standardized perioperative treatment, surgical debridement, and NPWT placement protocols were implemented for all enrolled patients. Clinical data including baseline demographic characteristics, systemic comorbidities, infection distribution, surgical incision selection, NPWT application frequency, intraoperative and postoperative complications, drainage duration, and hospitalization outcomes were systematically collected and descriptively analyzed.

**Results:**

The enrolled cohort consisted of 10 males and 5 females, with a mean age of 62.5 ± 16.9 years. Most patients presented with underlying systemic comorbidities, including hypertension (8/15) and diabetes mellitus (7/15), which were the most common concurrent diseases. The vast majority of cases (14/15) originated from mandibular dental lesions. Preoperative CT examination detected characteristic soft tissue gas accumulation in 7 patients (46.67%). All patients exhibited multi-space infection involvement, with the infratemporal space being the most commonly involved anatomical site, followed by the superficial temporal space and masseteric space. Individualized surgical incisions were formulated according to the infection scope, including single temporal incision (3 cases), single submandibular incision (1 case), and combined temporal and submandibular incision (11 cases). In terms of NPWT application, 8 patients received one-stage debridement and NPWT treatment, 6 patients underwent secondary debridement and NPWT replacement due to residual necrotic fascia, and 1 patient received three rounds of surgical debridement. The mean NPWT drainage duration was 12.47 ± 5.79 days, the mean dressing change frequency was 3.53 ± 0.64 times, and the average total hospitalization length was 23.40 ± 10.90 days. No severe intraoperative or postoperative complications, in-hospital mortality, or recurrent infection occurred in all patients.

**Conclusion:**

NPWT appears to possess favorable clinical operability and acceptable perioperative safety in the management of rare odontogenic ANF. Individualized incision planning tailored to the anatomical extent of infection may reduce surgical trauma and yield improved postoperative cosmetic results. Regular NPWT dressing changes combined with staged repeated debridement represent essential procedural measures suitable for the dense anatomical properties of craniomaxillofacial fascia. This single-arm case series only preliminarily demonstrates the potential clinical feasibility of NPWT for odontogenic ANF, with satisfactory short-term wound healing and infection control observed in the cohort. Owing to the limitations inherent to a non-controlled retrospective design, large-scale multicenter prospective controlled trials are still needed to verify its long-term efficacy and establish standardized application protocols.

## Introduction

 Necrotizing fasciitis (NF) is a rare, rapidly progressive, and life-threatening necrotizing soft tissue infection characterized by extensive necrosis of fascial planes and adjacent soft tissues [1, 2]. This aggressive infectious disease predominantly involves the trunk, extremities, and perineum, with high clinical mortality. In recent years, the incidence of head and neck NF has been gradually reported in clinical practice [3, 4]. Nevertheless, existing literature mainly focuses on cervical NF, while craniomaxillofacial NF is extremely rare in clinical settings.

Odontogenic infection is the dominant etiology of head and neck NF. Without timely and standardized surgical intervention, odontogenic inflammatory lesions can rapidly progress along fascial planes, potentially inducing severe complications such as airway obstruction, descending mediastinitis, and systemic sepsis [5–7]. Different from caudal cervical spreading infection, odontogenic infection can spread upward along the deep facial fascial system. We refer to this cranially extending necrotizing fasciitis as ascending necrotizing fasciitis(ANF), in contrast to descending infections. Despite lacking official nomenclature recognition, the term well illustrates the clinical features of the condition. ANF is a scarcely reported NF subtype with limited clinical experience and unified treatment criteria [8–10].

Odontogenic ANF frequently involves multiple deep facial spaces, including the buccal, masseteric, pterygomandibular, infratemporal, superficial temporal, and deep temporal spaces, and may further invade the galea aponeurotica in advanced progressive stages. The deep facial spaces possess irregular anatomical morphology and are densely filled with muscular and connective tissues. Such complex anatomical characteristics greatly increase the difficulty of thorough debridement and unobstructed drainage via conventional surgical methods, often resulting in persistent residual inflammation, prolonged hospitalization, and even facial functional impairment [11].

Negative pressure wound therapy (NPWT), also known as vacuum-assisted closure (VAC), is a mature wound treatment technique that promotes granulation tissue proliferation and accelerates inflammatory exudate drainage through continuous stable micro-negative pressure suction. It has been widely applied in the management of traumatic wounds, diabetic foot ulcers, and various refractory soft tissue infections [12–14]. Our previous single-center study has confirmed the clinical practicability of NPWT in the treatment of cervical necrotizing fasciitis [15].

Compared with the cervical region, the craniomaxillofacial fascial system has unique anatomical particularities. The temporal fascia, masseteric fascia, and galea aponeurotica are denser and thicker, with tight adhesion to cranial and facial bony structures, resulting in limited soft tissue mobility and narrow, irregular dead spaces in deep facial infection lesions. These anatomical features bring great challenges to the precise placement of NPWT sponges and complete coverage of necrotic lesions. In addition, dense facial fascia is not prone to spontaneous fragmentation and shedding under negative pressure suction, which may affect the continuous cleaning of necrotic tissues.

To date, no clinical studies have systematically explored the clinical feasibility, operational key points, and safety of NPWT for odontogenic ANF. Given the extremely low incidence of odontogenic ANF, single-center acquisition of sufficient synchronous matched cases for controlled comparative research is difficult and impractical. Therefore, this study adopted a single-arm retrospective case series design to systematically summarize the clinical data of 15 consecutive odontogenic ANF patients treated with NPWT. This study mainly focused on the technical operability, perioperative safety, feasible surgical strategy, and preliminary short-term clinical outcomes of NPWT for ANF, aiming to provide empirical support for the standardized clinical application of NPWT in rare craniomaxillofacial necrotizing infections.

## Methods

### Study participants

This study adopted a single-center, single-arm, retrospective feasibility study design. A total of 15 consecutive patients diagnosed with odontogenic ANF who received standardized NPWT treatment at the 82nd Group Army Hospital of PLA from March 2016 to March 2024 were enrolled. Unified diagnostic criteria were strictly implemented: (1) confirmed odontogenic infectious focus as the primary etiology; (2) typical clinical manifestations including facial swelling, severe local pain, trismus, and systemic inflammatory response; (3) preoperative craniofacial CT imaging confirming fascial thickening, soft tissue edema, or pathognomonic gas accumulation in deep facial spaces; (4) intraoperative direct visualization: grayish-black necrotic fascia, dishwater-gray fluid, absence of frank pus, absent active bleeding on fascial cut surfaces, effortless blunt dissection of fascia, and positive finger test. Exclusion criteria included patients with non-odontogenic infections, single soft tissue abscess or cellulitis without fascial necrosis, and incomplete clinical data.

This study was approved by the Ethics Committee of the 82nd Group Army Hospital of PLA (Approval No. 2023008) and conducted in strict accordance with the Helsinki Declaration and its subsequent amendments. Given the retrospective nature and anonymous data processing of this study, the requirement for informed consent was waived.

### Treatment protocols

All enrolled patients received standardized unified perioperative management. After admission, routine physical examination, laboratory inflammatory biomarker detection, and craniofacial CT scanning were performed to clarify the scope of infection, involved fascial spaces, and the degree of soft tissue necrosis (Fig. 1). Empirical intravenous antibiotic therapy with meropenem (1 g per dose, administered every 8 h) was administered immediately after admission, and pus specimens were collected before antibiotic initiation for aerobic and anaerobic bacterial culture and drug susceptibility testing.

**Fig. 1:**
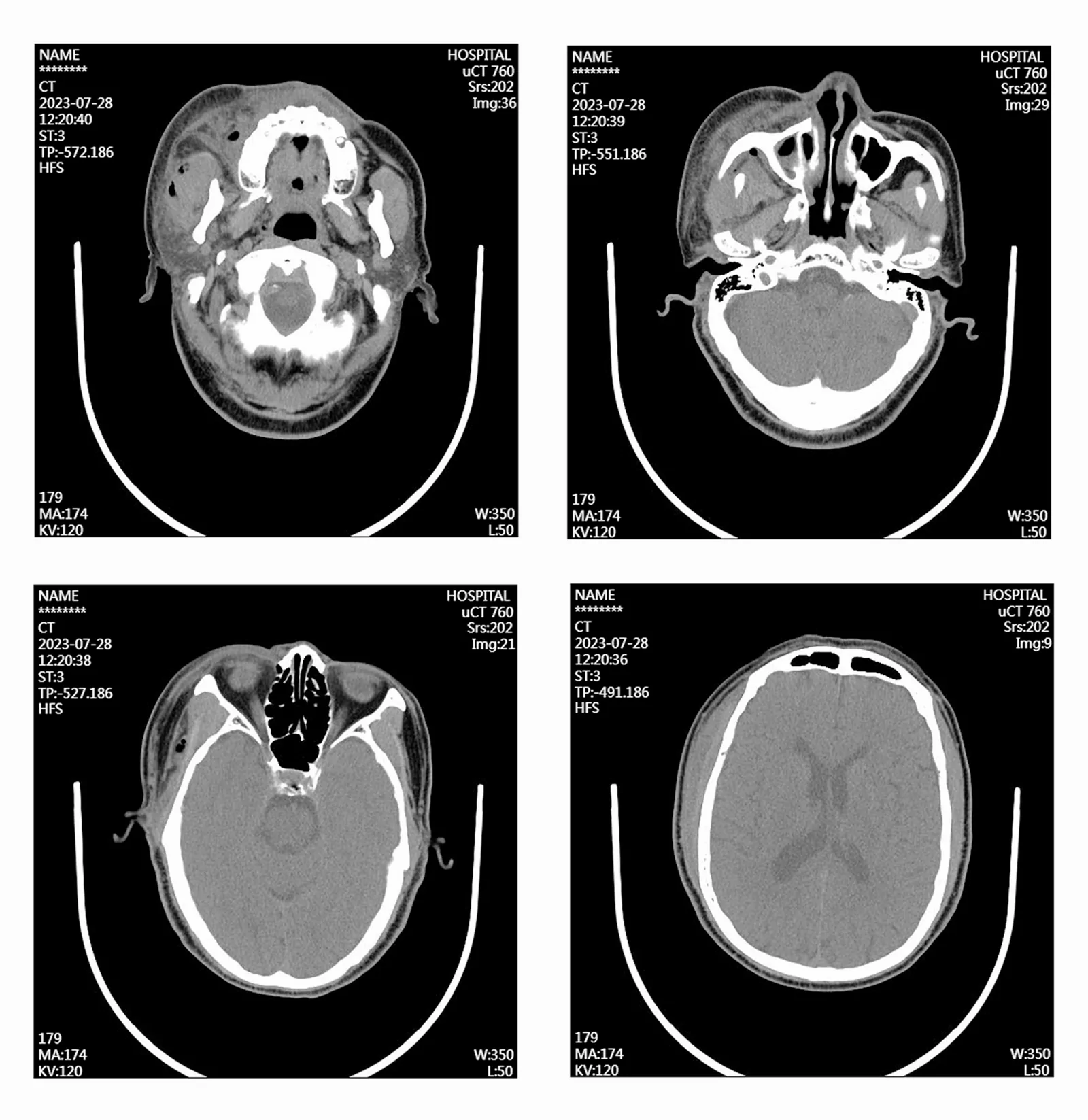
Axial CT image shows odontogenic ascending necrotizing fasciitis involving the buccal space, masseteric space, infratemporal space, and superficial temporal space

All patients underwent emergency surgical debridement and NPWT placement under general anesthesia within 6 h after admission. Individualized surgical incisions were designed according to the anatomical distribution and scope of infection: (1) single temporal hairline arc incision for single infections limited to supramandibular fascial spaces; (2) single submandibular incision for infections confined to low facial spaces; (3) combined temporal and submandibular through incision for extensive multi-space infections involving both upper and lower facial regions.

Intraoperatively, blunt dissection was performed along the natural fascial plane to fully expose all involved necrotic spaces. Thorough debridement was performed intraoperatively to remove as much necrotic fascia, inflammatory granulation tissue and purulent exudate as possible. The wound cavity was alternately irrigated with 3% hydrogen peroxide and normal saline until the cavity was clean. Subsequently, polyvinyl alcohol (PVA) NPWT foam dressings (Model: VSD-A-2, dimensions: 15 × 10 × 1 cm, Wuhan VSD Medical Science & Technology Co., Ltd., China) were trimmed according to the depth and volume the infection space. The trimmed foam dressing was tunneled into the lesion using hemostatic forceps, with its distal tip reaching the apex of the infected fascial space to fully cover the wound surface of the entire space. The foam dressing is closely attached to the incision and slightly extended outside the wound margin. This prevents premature wound closure that would hinder dressing removal, while facilitating real-time observation of negative pressure sealing status. Loose sutures are placed to approximate the wound and fix the foam sponge for proper wound compression. For adjacent fascial spaces, an alternative technique can be adopted: the foam dressing is cut from the center while retaining an intact root connection, allowing a single drainage tube to provide drainage for both spaces simultaneously. The wound and surrounding intact skin were completely sealed with semipermeable adhesive membrane (Fig. 2). Postoperatively, patients were admitted to the general ward. The drainage tube was connected to a central negative pressure system. A continuous stable negative pressure of 125 mmHg was maintained postoperatively, combined with intermittent 0.9% sodium chloride solution cavity irrigation to ensure unobstructed drainage.

**Fig. 2:**
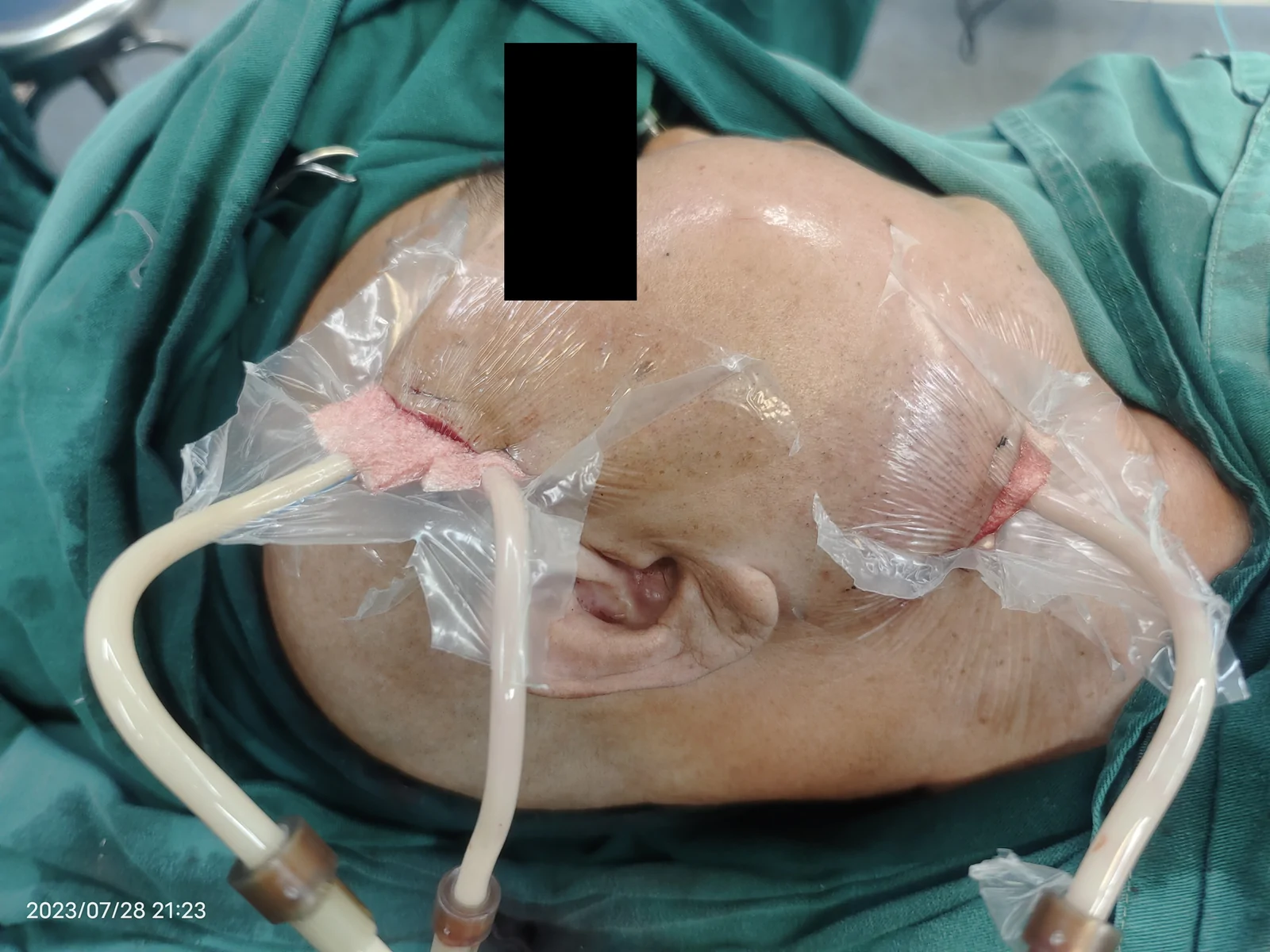
Custom-trimmed NPWT sponges are precisely placed to adapt to the irregular infected deep facial spaces

Postoperative routine monitoring of vital signs, drainage volume, and wound status was performed. Repeat craniofacial CT examination was conducted on postoperative day 3 to evaluate infection control and NPWT sponge positioning. According to the drainage clarity, wound granulation tissue growth, and local inflammatory control status, NPWT devices were replaced or removed 5–10 days after surgery. Secondary surgical debridement was performed for patients with persistent residual necrotic tissues or turbid purulent exudate. After NPWT removal, closed-suction drains were placed in the spaces with controlled infection, and the wounds were sutured. The drainage was maintained for 48 h before removal. Afterwards, the wounds healed naturally, followed by suture removal.

### Data collection and statistical analysis

Systematic collection and sorting of complete clinical data of enrolled patients were performed, including baseline demographic information, systemic comorbidities, pathogenic dental focus characteristics, distribution of involved fascial spaces, clinical symptoms, preoperative laboratory and imaging findings, surgical incision type, NPWT application and replacement frequency, intraoperative conditions, postoperative complications, NPWT drainage duration, and total hospitalization length.

All statistical analyses in this study adopted descriptive statistical methods. Quantitative data were expressed as mean ± standard deviation, and counting data were expressed as frequency and constituent ratio. No inter-group comparative statistical analysis was performed in this single-arm feasibility study.

## Results

### General baseline clinical characteristics

The clinical characteristics are summarized in Tables 1 and 2. A total of 15 patients with odontogenic ANF were enrolled in this study, including 10 males and 5 females, with a mean age of 62.5 ± 16.9 years (range: 24–88 years). Only 2 patients had no underlying systemic diseases, while the remaining 13 patients were complicated with chronic systemic comorbidities, including hypertension (8/15), diabetes mellitus (7/15), moderate anemia (3/15), and coronary heart disease (2/15).

**Table 1:** Clinical characteristics of the patients

Variable	Value
Sex, *n*	15
Male	10
Female	5
Mean age, years, mean ± SD	62.5 ± 16.9
Range	24–88
Source tooth site, *n*	
Mandibular third molar	4
Mandibular second molar	3
Mandibular first molar	1
Mandibular second premolar	1
Multiple contiguous mandibular odontogenic lesions	6
WBC count, ×10^9^/L, *n*	
> 10.00	11
> 20.00	6
CRP, mg/L	
Mean value	107.50 ± 91.99
Range	24.30–320.00
Surgical incision type, *n*	
Single temporal incision	3
Single submandibular incision	1
Combined temporal and submandibular incision	11
NPWT application frequency, *n*	
Once	8
Twice	6
Three times	1
Mean duration of NPWT, days, mean ± SD	12.47 ± 5.79
Mean total hospitalization length, days, mean ± SD	23.40 ± 10.90

*Abbreviations* *NPWT * negative pressure wound therapy, *WBC * white blood cell, *CRP * C-reactive protein

**Table 2:** Involved spaces of the 15 patients

Involved spaces	No.
Infratemporal space	15
Superficial temporal space	13
Masseteric space	13
Buccal space	10
Pterygomandibular space	9
Submandibular space	5
Parapharyngeal space	3
Galea aponeurotica	2
Deep temporal space	2
Parotid space	1
Submental space	1

Odontogenic pathogenic analysis showed that single cariogenic or periapical diseased teeth were identified in 9 cases, with the third molar being the most common pathogenic tooth (4/9), followed by the second molar (3/9), first molar (1/9), and second premolar (1/9). The remaining 6 cases were attributed to multiple consecutive mandibular dental lesions with undetectable single independent pathogenic teeth. Notably, 14 of the 15 cases originated from mandibular dental lesions, and only 1 case was suspected to be associated with right maxillary third molar disease. This patient had a history of right maxillary third molar tooth extraction and subsequently developed symptoms of facial infection. However, there was also severe periodontitis in right mandibular third molar on admission.

All patients presented with typical clinical symptoms of facial swelling, severe local pain, and limited mouth opening. Additional clinical manifestations included odynophagia (3/15) and dyspnea (2/15), while only 2 patients had fever symptoms. Preoperative laboratory examination showed significant systemic inflammatory activation: 11 patients (73.33%) had elevated white blood cell (WBC) count (> 10 × 10^9^/L), among whom 6 patients (40.00%) had WBC count exceeding 20 × 10^9^/L. The mean neutrophil percentage was 82.81 ± 8.47%, and the mean C-reactive protein level was 107.50 mg/L (range: 24.30–320.00 mg/L). Preoperative craniofacial CT scanning detected pathognomonic soft tissue gas accumulation in 7 patients (46.67%), which was consistent with the imaging diagnostic characteristics of necrotizing fasciitis.

### Involved fascial spaces and surgical characteristics

In addition to the fascia overlying the masticator muscles, intramuscular fascia is present within the muscle substance. Infections involving these intramuscular fascial layers are categorized into the corresponding masticator spaces: infections of the fascia within the masseter muscle belong to the masseteric space, whereas fascial infections of the pterygoid and temporalis muscles correspond to the pterygomandibular space and deep temporal space, respectively. All enrolled patients presented with simultaneous involvement of four or more deep facial fascial spaces. The infratemporal space was involved in all 15 cases, being the most susceptible anatomical site, followed by the superficial temporal space (13/15) and masseteric space (13/15). Galea aponeurotica involvement was observed in 2 cases, and deep temporal space involvement was confirmed in 2 cases. According to the infection scope, 9 patients had single supramandibular space infection, and 6 patients had combined craniofacial and upper cervical infection. The lesion side distribution was relatively balanced, with a left-to-right ratio of 7:8. Six patients had received incomplete small-incision drainage treatment in external medical institutions before admission, with residual persistent infection.

All patients successfully completed surgical debridement and NPWT device placement under general anesthesia without intraoperative complications. Postoperatively, all 15 patients were transferred back to the general ward, and there was no need for ICU support. According to individual infection characteristics, 3 patients received single temporal incision, 1 patient received single submandibular incision, and 11 patients received combined temporal and submandibular incision. In terms of NPWT treatment sessions, 8 patients achieved satisfactory intraoperative debridement and wound condition, with only one session of NPWT application required, and healthy granulation tissue covered the wound cavity during device removal (Fig. 3). Six patients presented with persistent local inflammatory exudation and residual necrotic fascia after the first treatment, requiring secondary debridement and NPWT device replacement. One patient received three rounds of sequential surgical debridement due to repeated local tissue necrosis, and no adverse events occurred during the treatment process.

**Fig. 3:**
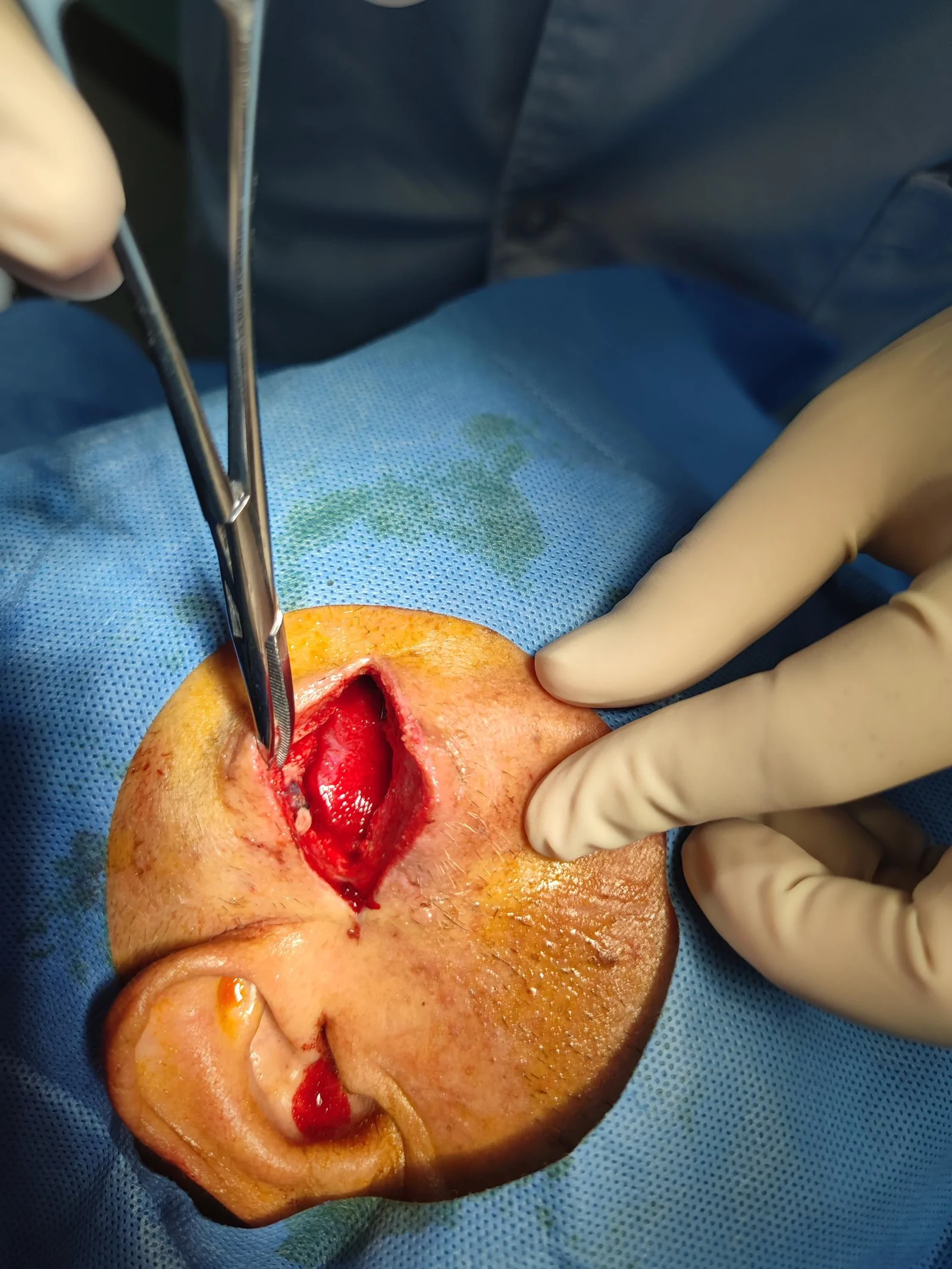
Fresh, healthy granulation tissue covers the wound cavity when the NPWT device is removed, indicating satisfactory wound regeneration

### Treatment outcomes and clinical prognosis

The clinical outcome data of all 15 NPWT-treated patients were descriptively analyzed. The mean duration of NPWT continuous drainage was 12.47 ± 5.79 days, the mean frequency of postoperative dressing replacement was 3.53 ± 0.64 times, and the mean total hospitalization length was 23.40 ± 10.90 days. All patients achieved gradual wound healing and complete infection control after standardized NPWT treatment. No severe postoperative complications, in-hospital mortality, or short-term infection recurrence occurred during hospitalization. After NPWT removal, all wounds achieved smooth staged healing without residual pus cavity or persistent inflammatory lesions (Fig. 4).

**Fig. 4:**
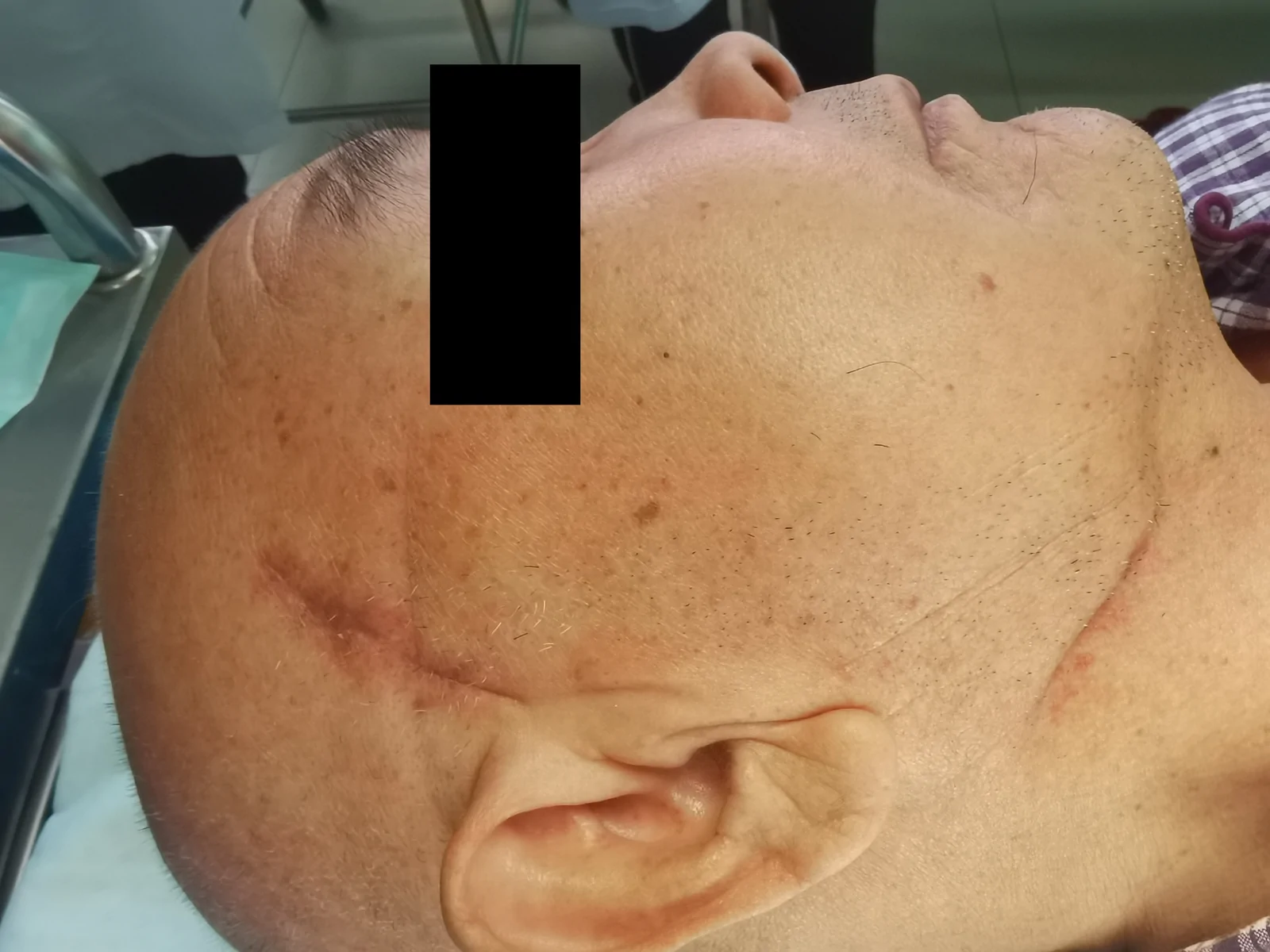
The wound achieves smooth primary healing after NPWT removal without residual infection or deformity

### Bacterial culture results

Valid aerobic and anaerobic bacterial culture results were obtained in only 3 patients, including *Streptococcus intermedius* (1 case), *Staphylococcus aureus* (1 case), and *Serratia liquefaciens* (1 case). The extremely low positive culture rate was mainly attributed to the universal empirical antibiotic treatment received by most patients before admission and the rigorous culture conditions required for anaerobic pathogens.

## Discussion

Necrotizing fasciitis is a life-threatening progressive necrotizing soft tissue infectious disease with a long clinical recognition history. Since the earliest description by Hippocrates, it has been named phagedaenic ulcers, streptococcal gangrene, and gas gangrene in different historical periods [16–18]. In 1952, Wilson formally proposed the unified nomenclature of “necrotizing fasciitis”, which has been widely recognized and adopted globally [19]. At present, NF is uniformly classified as a critical subtype of necrotizing soft tissue infection (NSTI) due to the consistent core treatment principles of various necrotizing soft tissue infections [18, 20, 21]. NF primarily occurs in the extremities, abdominal wall, and perineum, while head and neck NF are relatively rare in clinical practice, with odontogenic infection as the dominant pathogenic inducement [6, 22]. Existing clinical research on head and neck NF mainly focuses on descending cervical NF, due to its high risk of fatal complications such as acute airway obstruction and descending mediastinitis [23]. In contrast, odontogenic ANF, a rare variant characterized by upward fascial spread, is vastly underreported in current literature, with no standardized mature treatment system formed. The low clinical recognition and insufficient research of ANF are mainly attributed to two factors: firstly, ANF has a low acute mortality rate compared with cervical NF, leading to insufficient clinical attention and easy misdiagnosis as common severe facial cellulitis; secondly, the complex multi-layered and concealed anatomical structure of deep facial fascial spaces increases the difficulty of complete intraoperative exposure and accurate early diagnosis.

The craniomaxillofacial fascial system is a continuous anatomical extension of the cervical fascia [24]. The superficial layer of the deep cervical fascia divides into two laminae at the inferior border of the mandible. The external lamina courses superficially along the mandible, encircles the parotid gland and masticatory muscles laterally, and forms the parotid-masseteric fascia. This fascia continues across the zygomatic arch and transitions into the temporal fascia. The internal lamina runs medially and posteriorly deep to the medial pterygoid muscle, and ultimately fuses with the fascia covering the styloglossus muscle and its surrounding tissues. The temporal fascia consists of superficial and deep layers, both of which connect to the galea aponeurotica. In cases of odontogenic necrotizing fasciitis, infection may spread superiorly along these fascial planes and eventually involve the galea aponeurotica. We refer to this ascending infection as ANF. In terms of clinical manifestations, necrotizing fasciitis of the deep fascial fascia also presents as gray black necrosis, foul smelling secretions, and positive finger test.

Deep facial space infection is a rare form of craniocervical infection with a low incidence and limited specialized research coverage in existing literature [25]. Different from the loose and extensible cervical fascia, the temporal fascia, masseteric fascia, and galea aponeurotica are dense, thick, and tightly adhered to facial bony structures, forming multiple closed, irregular deep facial spaces. Once infection occurs, the dense fascia is difficult to spontaneously detach and drain necrotic tissues, easily leading to residual infection and persistent repeated inflammation, which contributes to prolonged hospital stays and may further induce severe complications such as osteomyelitis and intracranial infection. Traditional open surgical drainage relies on passive gravity drainage and frequent manual dressing changes. For multi-space deep facial infections, combined temporal and submandibular incisions are commonly adopted for vertical penetrating drainage; however, this conventional surgical approach often fails to resolve the dilemma of inadequate drainage. Although coracoid process resection has been performed in individual cases to facilitate thorough drainage and yield favorable therapeutic outcomes, this invasive procedure causes irreversible and substantial tissue damage [11]. Our team has previously conducted preliminary research on the application of NPWT in cervical necrotizing fasciitis and achieved promising clinical results [15]. On this basis, this study further explored the application value of NPWT in craniomaxillofacial odontogenic ANF, focusing on the technical operability, individualized surgical strategy, and clinical safety of this technique in rare deep facial necrotizing infections.

This single-arm case series summarized the clinical experience of 15 ANF patients treated with NPWT. The study found that mandibular dental lesions accounted for 93.33% of all cases, which fully reflects the anatomical characteristics that mandibular odontogenic infection is prone to spread upward along deep facial fascial planes to induce ANF. Different from cervical NF, craniomaxillofacial ANF requires relatively repeated NPWT replacement and staged debridement. Intraoperative observation confirmed that the dense facial fascia necrotic tissues are not easily fragmented and discharged through negative pressure suction, and residual necrotic fascia often adheres to deep tissues. This indicates that routine NPWT replacement and repeated intraoperative debridement are necessary operational procedures adapted to the anatomical characteristics of craniomaxillofacial ANF, and the treatment cycle needs to be individualized according to intraoperative conditions and postoperative drainage status.

Individualized incision selection merits further clinical exploration regarding NPWT application in patients with ANF. Based on the anatomical scope of infection and NPWT placement accessibility, three feasible incision modes were summarized in this cohort. For single supramandibular space infections, single concealed temporal hairline incision may meet the drainage needs of multiple deep facial spaces through the gap around the zygomatic arch. This incision has the advantages of concealed scar, less soft tissue trauma, and avoidance of submandibular cosmetic defects. In our case series, three out of 15 patients achieved satisfactory infection control with this incision. However, the selection of this incision is limited by the medial space of the zygomatic arch. The space extending from the temporal region to the infratemporal and masticator spaces inside the zygomatic arch is confined, and sometimes it is even insufficient to accommodate NPWT dressings. Accordingly, a sole temporal incision is only appropriate for carefully selected patients. Likewise, a simple submandibular incision cannot achieve drainage of the temporal region through the zygomatic arch, and it is only applicable when infection is confined to the spaces beneath the zygomatic arch. For extensive multi-space infections involving both upper and lower facial regions, combined temporal and submandibular through incision is required to form a smooth drainage channel and ensure thorough infection control. The above individualized incision strategies can provide practical technical reference for clinical ANF surgical treatment. Nevertheless, our surgical selection protocols represent single-center experience only and should not be regarded as general therapeutic guidelines.

Currently, two mainstream NPWT dressings are widely applied in clinical practice: polyurethane (PU) foam and PVA foam, which possess distinct physical characteristics and clinical indications. PU foam features large pore size and high permeability, which favors adequate drainage of highly exudative and acutely infected wounds. However, it has inherent drawbacks when applied to deep maxillofacial fascial space infections. The large porous structure easily allows ingrowth of newly formed granulation tissue, inevitably causing mechanical tearing and bleeding during dressing removal. Moreover, deep and narrow maxillofacial dead spaces require dressings with reliable tensile resistance during implantation and extraction, whereas conventional PU foam lacks sufficient structural toughness for such complex anatomical scenarios. In comparison, PVA foam has dense, fine and smooth surface pores, which effectively prevent excessive granulation tissue ingrowth and eliminate secondary mechanical trauma and bleeding during dressing replacement. Additionally, PVA material demonstrates superior hydrophilicity, elasticity and tensile strength, making it more adaptable to deep, irregular, and confined maxillofacial wound cavities. For these reasons, PVA foam was selectively adopted as the primary dressing in the present standardized NPWT protocol. Nevertheless, the small pore size of PVA foam theoretically increases the risk of drainage tube obstruction by viscous exudates and tissue debris. To address this potential problem, intermittent postoperative normal saline irrigation was routinely performed in all patients. With this auxiliary measure, no drainage blockage occurred during the entire treatment course.

Compared with conventional open wound management, NPWT may offer distinct advantages in the standardized treatment of complex odontogenic ANF. First, the continuous closed negative pressure system sustainably eliminates inflammatory exudates, necrotic debris, and bacterial metabolites from deep layered fascial spaces, effectively reducing local bacterial burden and halting the progressive spread of necrotizing infection. This approach fundamentally overcomes the limitations of conventional therapy, including inadequate passive drainage and persistent wound exposure. Second, sustained negative pressure stimulation ameliorates local soft-tissue microcirculation and promotes orderly granulation tissue proliferation, thereby shortening wound healing time and reducing overall hospitalization length. Third, the closed drainage system eliminates frequent manual dressing changes and mechanical irritation to delicate maxillofacial soft tissues, substantially alleviating postoperative pain and minimizing iatrogenic secondary injury. Furthermore, the fully sealed wound environment isolates external bacterial contamination, decreasing the incidence of secondary wound infection, abscess recurrence, and progressive soft tissue necrosis in severe ANF patients. Notably, uncontrolled anaerobic infection is widely recognized as a relative contraindication for NPWT. The present study further confirms the feasibility of early NPWT intervention for severe maxillofacial necrotizing soft tissue infections. Nevertheless, NPWT possesses inherent limitations and clinical application constraints that cannot be overlooked in ANF treatment. First, the deep, irregular, and concealed anatomical features of maxillofacial fascial spaces increase the technical difficulty of precise and complete NPWT sponge placement. Second, the irregular facial contour complicates peripheral sealing of the semipermeable membrane, which may compromise negative pressure stability and drainage efficiency. Third, NPWT occludes direct visual observation of the wound bed; the dynamic status of infection control can only be comprehensively evaluated through clinical manifestations and laboratory examinations rather than real-time intuitive observation. In addition, NPWT consumables and equipment impose higher economic costs than traditional dressings, and standardized application requires professional real-time monitoring of negative pressure value, sealing integrity and drainage status, thereby increasing clinical nursing workload. Accordingly, specialized systematic training is essential to ensure standardized and safe NPWT implementation in complex maxillofacial necrotizing infections.

In terms of clinical safety and short-term prognosis, all 15 patients in this cohort completed NPWT treatment smoothly without severe perioperative complications or mortality. The standardized NPWT treatment mode effectively avoids the frequent wound exposure and repeated mechanical irritation caused by high-frequency manual dressing changes in traditional treatment, reduces the risk of secondary wound infection, and provides a stable microenvironment for wound granulation tissue regeneration. The preliminary short-term clinical outcomes of this cohort verify the good safety and clinical feasibility of NPWT for odontogenic ANF. Although the number of case series is relatively small, compared with the traditional drainage approaches reported in previous literature, this preliminary study has already demonstrated favorable clinical outcomes. Nevertheless, larger sample-sized data are needed to confirm these findings. Furthermore, cervicofacial NF exhibits greater clinical complexity than routine pyogenic infections, and the proposed therapeutic strategy is equally effective for common deep facial space infections.

The limitations of this study must be objectively acknowledged. This is a single-center, single-arm retrospective feasibility study without a synchronous control cohort, so the current data can only confirm the clinical feasibility and short-term safety of NPWT for ANF, and cannot quantitatively verify the superiority of NPWT compared with conventional open drainage. Given the extremely low incidence of odontogenic ANF, it is difficult to collect sufficient baseline-matched synchronous control cases in a single center for standardized controlled research. In addition, the low positive rate of bacterial culture fails to provide sufficient bacteriological etiological evidence, and this study lacks long-term follow-up evaluation of facial function and cosmetic prognosis. Therefore, multi-center, large-sample, prospective controlled studies are still needed in the future to further supplement and verify the long-term clinical efficacy and standardized application specifications of NPWT for odontogenic ANF.

In conclusion, NPWT appears to offer acceptable clinical operability and relatively reliable perioperative safety for the treatment of rare odontogenic ANF. For properly selected patients, individualized surgical incision planning based on the anatomical range of infection may reduce surgical trauma and achieve better postoperative cosmetic results. Regular NPWT dressing replacement combined with staged repeated debridement represents a technically necessary regimen tailored to the dense and tough anatomical features of craniomaxillofacial deep fascia. Within the limitations of this single-center retrospective case series, our results only preliminarily support the potential clinical value of NPWT for odontogenic ANF. High-level multicenter prospective controlled trials are still required to establish its definitive efficacy before forming unified clinical application guidelines.

## Data Availability

All data generated or analyzed during this study are included in this published article.

## Abbreviations

NF: Necrotizing fasciitis
ANF: Ascending necrotizing fasciitis
NPWT: Negative pressure wound therapy
CT: Computed tomography
VAC: Vacuum-assisted closure
NSTI: Necrotizing soft tissue infection
PVA: Polyvinyl alcohol
PU: Polyurethane
